# Effects of social exclusion on following the gaze of others

**DOI:** 10.1111/bjop.70034

**Published:** 2025-10-06

**Authors:** Ala Yankouskaya, Claudia Salera, Marianna Constantinou, Anna Pecchinenda

**Affiliations:** ^1^ Department of Psychology Bournemouth University Poole UK; ^2^ SmArt Lab, IRCCS Santa Lucia Foundation Rome Italy; ^3^ College of Science and Engineering Hamad Bin Khalifa University Doha Qatar; ^4^ Department of Psychology Sapienza University of Rome Rome Italy

**Keywords:** Cyberball, emotional expression, face, gaze following, joint attention, ostracism, social exclusion

## Abstract

Evidence shows that social exclusion motivates to paying attention to the situation to reconnect with others or to protect oneself from further exclusion. However, it is unclear how social attention is affected by who offers an opportunity to reconnect. Two studies filled this gap by assessing whether being excluded affects our propensity to share attention with another individual (seen or novel) with a happy or a neutral expression. Findings show a significant three‐way interaction with differences in gaze cueing between groups only for seen faces with a neutral expression. Gaze‐cueing effects for seen (excluders) faces with a neutral expression occurred in 73% of socially excluded individuals – this was 33% for seen (includers) faces for socially included. There were no differences in gaze cueing for novel faces with happy or neutral expressions. In Study 2, social information about faces was learned without direct exclusion. Here, the proportion of participants showing the effect observed in Study 1 and the associations between gaze cueing and emotional expressions differed. In line with the social monitoring system theory, individuals in the immediate aftermath of exclusion remain socially engaged, displaying a dual attentional strategy: vigilance towards the excluder and openness to affiliative signals from novel others.

## BACKGROUND

The need to belong is one of the basic human needs: when threatened by acts of social exclusion – whether intentionally or not – it has harmful consequences for the individual (Baumeister & Leary, 1995). According to the temporal need‐threat model (Williams, 2009), the individual's reaction to being socially excluded or ignored – i.e., being ostracised – unfolds in three stages. In the *reflexive stage*, being socially excluded is experienced as social pain, which commands the individual to pay attention to the social situation. Importantly, the pain of social exclusion shares neural substrates and processes with physical pain (Eisenberger & Lieberman, 2004). Perhaps due to this quality, the reflexive response to ostracism does not depend on the individual's characteristics, as it seems to be a relatively universal reaction to the detection of social exclusion. What characterises the next stage of the response to ostracism – the *reflective stage* – is an attempt to cope with the social exclusion, either by trying to be re‐included if possible, or by protecting oneself from further exclusion. According to the model, what happens at this stage depends on individual characteristics and on our need to belong, self‐esteem, as well as control and meaningful existence. Finally, the third and last stage – *the resignation stage* – refers to the long‐lasting, devastating effects of ostracism, characterised by feelings of alienation, low self‐worth, and helplessness. Here we focus on the immediate effects that being socially excluded has on attention to social information (Study 1), and more specifically on the propensity to follow the gaze of another person and share the focus of attention (i.e., joint attention).^1^ In addition, we assess whether social learning – that is, having just acquired information related to social exclusion about an individual (Study 2) – is sufficient to affect gaze following. This would help establish whether experiencing social exclusion is necessary to affect attention to social information.

Social exclusion in the laboratory can be successfully manipulated using the Cyberball task (Williams et al., 2000; Williams & Jarvis, 2006), a virtual ball‐tossing game involving three players: the participant and two virtual players. In the inclusion condition, the number of tosses between players is equal, whereas in the exclusion condition, the participant receives only the first two tosses, and for the rest of the game, the two other players exchange ball tosses. This task has been shown to successfully manipulate ostracism, with a large effect size (Hartgerink et al., 2015). Following the gaze of others and showing joint attention can be assessed with the gaze‐cueing task (Driver IV et al., 1999; Friesen & Kingstone, 1998). The task consists of presenting a central face looking left or right, which is not predictive of target location, followed by a target presented left or right of fixation. Participants respond to the target based on some stimulus characteristics. The typical finding is of faster responses to targets presented at the cued location (i.e., the location looked at by the face cue) compared to targets presented at the spatial location opposite to that cued by the face (McKay et al., 2021). This effect is known as the gaze‐cueing effect, and it is attributed to the gaze direction of another individual being an important social signal used to make inferences about the mental state and the focus of attention of others (Baron‐Cohen, 1995; Driver IV et al., 1999; Gallagher & Frith, 2003). Importantly, neuroimaging evidence shows an overlap between the neural bases of social rejection and our ability to attribute mental states to others (Sahi & Eisenberger, 2021). The effects of this overlap on social behaviours were evident during the COVID‐19 pandemic when social isolation affected hard‐wired cognitive mechanisms critically supporting the establishment of social interactions and relationships among individuals. In fact, during the lockdown period, people showed a hyper‐sensitivity to social cues (and stronger gaze cueing), probably due to the strive to reconnect with others (Dalmaso et al., 2021).

To date, the immediate effect of being ostracised on joint attention – one of the key abilities for social cognition and interacting with others – has been scarcely investigated. Namely, Capellini et al. (2019) manipulated social exclusion using the Cyberball task and assessed its effect on orienting attention to gaze cues (Study 1). Participants completed one block of 128 trials in which a face with straight gaze (i.e., the pre‐cue) was first presented for 900 ms, followed by the same face with averted gaze (i.e., the gaze‐cue) for 200 ms (SOA), after which the target (L or T) appeared next to the face cue, on the left or right of the screen. Cues were not predictive of target location, and participants responded based on target identity. They found reduced gaze‐cueing effects for the social exclusion group. This reduced cueing effect was not present for arrow cues (Study 2), suggesting that social exclusion affects orienting to social cues rather than to directional cues in general. This is important as it is argued that orienting attention by following the gaze of another person is a unique form of social attention (Friesen & Kingstone, 1998), which relies on neural circuits that are partially segregated from those involved in orienting attention by non‐social cues (Salera, Boccia, & Pecchinenda, 2024; Salera, Yankouskaya, et al., 2024). More recently, Yang et al. (2024) have shown that this effect is specific to the face of the excluder. They also used the Cyberball to induce social exclusion, followed by the gaze‐cueing task. However, they assessed the role of SOA (200 ms vs. 700 ms) and whether social exclusion affects joint attention with other individuals in general or only for the one responsible for the social exclusion (i.e., the excluder). This was done by presenting the three players' avatars in the Cyberball task, with three face pictures, two strangers' faces and the participant's face. The two strangers' faces used in the Cyberball were then used in the gaze‐cueing task together with two novel faces. Participants completed 384 trials of the gaze‐cueing task in which a central face with averted gaze (i.e., the gaze‐cue) was presented for 200 or 700 ms (SOA varied randomly), followed by the target (a black dot) presented left or right of the face, to which participants responded based on the spatial location (left/right). Findings showed no effects of being ostracised on gaze cueing at short SOA (200 ms) and reduced gaze cueing only for the specific excluder faces when assessed at long SOA (i.e., 700 ms). Therefore, both studies point to social exclusion reducing gaze‐cueing effects, especially with the social excluders. In contrast, Wilkowski et al. (2009) found enhanced gaze‐cueing effects for individuals who were asked to write about a past occasion in which they felt socially rejected and excluded. They used a face with a straight gaze (the pre‐cue) for 500 ms, followed by the face with averted gaze for 50 or 600 ms (Study 2). Importantly, and regardless of the methodological differences between these studies (i.e., SOAs, pre‐cue), in all cases neutral faces were used, and it is unclear whether joint attention is also reduced when individuals – especially the excluders – signal affiliative intents by smiling. For instance, there is evidence that ostracised individuals are faster at detecting smiling faces in a visual search task (DeWall et al., 2009). In addition, recent evidence using experience sampling from real‐life situations (Büttner et al., 2024) shows that ostracised individuals report both pro‐social, approach tendencies as well as anti‐social, avoidance tendencies, depending on how much their need to belong has been threatened.

Based on the evidence described above, one could argue that the immediate experience of being socially excluded is linked to a reduction in the tendency to join attention with others (i.e., Capellini et al., 2019; Yang et al., 2024) but retrieving information of social exclusion from memory leads to an increment in the tendency to join attention with others (i.e., Wilkowski et al., 2009). Importantly, information about an individual can be acquired (i.e., social learning, see Lieberman et al., 2002) by direct experience of being socially excluded as in Yang et al. (2024), by experiencing pro‐social or anti‐social interactions (Hudson et al., 2012), or by learning written information about them (Dalmaso et al., 2012, 2014). However, in these cases, the direction of the effects of the learned knowledge on gaze cueing may vary. More specifically, Hudson et al. (2012) manipulated the social disposition of face identities implicitly by presenting short video clips of two faces, engaging in prosocial (direct gaze and smile) or anti‐social disposition (averted gaze and angry) towards the observer. They then used these faces in the gaze‐cueing task and found enhanced gaze‐cueing effects for prosocial faces. In contrast, Dalmaso et al. (2012) asked participants to read fictive curricula vitae of high or low social status jobs for faces of different individuals before using these faces in the gaze‐cueing task. They found an enhanced gaze‐cueing effect for high‐status faces. Similarly, Carraro et al. (2017) found larger gaze‐cueing effects for faces associated through learning with antisocial behaviour. In addition, Dalmaso et al. (2014) showed that the learned social information about faces is extracted quickly when these faces are used in the gaze‐cueing task, and its effect on joint attention occurs already at short stimulus onset asynchrony (SOA 200 ms).

Here, we used the gaze‐cueing task to assess to what extent the tendency to share attention with a person already seen (i.e., the excluder for the exclusion group and the includer for the inclusion group) or with a novel person occurs shortly after having experienced social exclusion (Study 1) and after having learned episodic information related to social exclusion about a person (Study 2). In Study 1, social exclusion was experienced immediately before the gaze‐cueing task while playing the Cyberball. In Study 2, a written description of an episode of social exclusion was learned for certain individuals before the gaze‐cueing task. In both studies, we presented happy and neutral faces of seen and novel individuals, and we used a pre‐cue as it allows establishing eye contact with the observer. As direct gaze may signal the intention to communicate and interact, and a happy face can signal affiliation, both direct gaze and happy expression can communicate approach tendencies (e.g., Adams & Kleck, 2005). It is important to note that using the pre‐cue to establish eye contact may also help to minimise the effects that social exclusion has on the perception of averted gaze (i.e., cone of gaze) as ostracised individuals tend to misinterpret a wider range of gaze deviations (i.e., a wider cone of gaze; Lyyra et al., 2017 or a narrower cone of gaze; Syrjämäki et al., 2020). That is, presenting a face with direct gaze first and then with averted gaze entails implied motion, which makes this social signal less ambiguous. Indeed, it is well documented that typically, gaze‐cueing effects are larger when using a pre‐cue face (McKay et al., 2021). Finally, in keeping with two of the previous studies (Yang et al., 2024 used an SOA of 700 ms; Wilkowski et al., 2009 used an SOA of 600 ms) but also because gaze‐cueing effects are small at short SOAs, peak at medium SOAs, and then start to reduce from SOAs of 800 ms onward (McKay et al., 2021), we used an SOA of 700 ms.

To sum up, in Study 1, we used the Cyberball task to manipulate social exclusion from specific individuals by presenting the two avatar players with pictures of two female faces. Next, we assessed gaze following using a gaze‐cueing task, in which participants were presented with the two faces previously seen in the Cyberball game (excluder faces for the group assigned to social exclusion, and includer faces for the group assigned to social inclusion) and with two new faces not encountered previously. The faces used in the gaze‐cueing task were presented first with straight gaze – that is, gazing at the observer to establish eye contact – and then with averted gaze, either smiling or maintaining a neutral expression. Considering the overlap between the neural bases of social rejection and mentalising (Sahi & Eisenberger, 2021), we anticipate that if being socially excluded motivates us to protect ourselves from further exclusion and affects gaze cueing depending on prior familiarity with the face (i.e., having encountered the individual who excluded us in the Cyberball, then in line with Yang et al., 2024) gaze‐cueing effects should be smaller only for the excluder face with a neutral expression. If the effect of being excluded on social attention is general and not specific to the excluder, then there should be smaller cueing effects for neutral faces, for both excluder and novel faces (Capellini et al., 2019). However, if the immediate effect of being socially excluded by someone is to follow their gaze when they offer the opportunity to reconnect by smiling (for a similar argument, see DeWall et al., 2009 for selective attention; Philipp et al., 2021 for smiling), then gaze‐cueing effects should be larger for the excluder face when presented with a happy expression. Indeed, there is evidence that when using emotional faces in the gaze‐cueing task, contextual factors (e.g., Pecchinenda et al., 2008; for a review, see Dalmaso et al., 2020) can enhance gaze‐cueing effects for emotional faces. Finally, if these effects rely on experiencing social exclusion, then they should be present in Study 1 but not in Study 2.

## METHOD

We report how we determined our sample size, all data exclusions (if any), all manipulations, and all measures in the studies. Data collection for the two studies took place from October 2024 to January 2025. Data and study materials are available at the Open Science Framework (OSF) at the following link: https://osf.io/tmf4p/?view_only=6a5bfdddedc74e0ba1a9e4ec9d7ca5cf.

### Study 1

#### Participants

Ninety‐four university students (75 F; 19 M; age in years *M* = 20.76; SD = 2.5) volunteered to take part in exchange for course credit. Half were randomly assigned to the inclusion (*N* = 47, 10 M; age in years *M* = 20.66; SD = 1.29) and the other half to the exclusion (*N* = 47, 9 M; age in years *M* = 20.85; SD = 3.22) condition of the Cyberball task. A meta‐analysis of 120 Cyberball studies found that the smallest sample size required for a between‐group design, where the ostracism manipulation was successfully detected, was 24 participants (Hartgerink et al., 2015). For the present study, we calculated the required sample size using effect sizes from previous research with a similar experimental design (Bernstein & Claypool, 2012), applying G*Power (Faul et al., 2009). To achieve 95% power with an alpha level < .05 and an effect size of *f* = 0.75 in fixed‐effects omnibus, one‐way ANOVA will require 26 participants in total to detect differences in the magnitude of the gaze‐cueing effect.^2^ All participants gave their informed consent, which was obtained according to the Declaration of Helsinki (1991). The study had received approval from the institutional review board (approval n. 0000867). Participants had normal or corrected‐to‐normal vision and were naïve to the purpose of the study.

#### Questionnaires

Participants completed the Italian translation of the Need‐Threat Scale (NTQ; Williams, 2009) used by Capellini et al. (2019), assessing participants' satisfaction levels for belongingness (e.g., “I felt rejected”), self‐esteem (e.g., “I felt liked”), control (e.g., “I felt powerful”), and meaningful existence (e.g., “I felt invisible”) on a scale from 1 (not at all) to 5 (completely); the Italian version of the Interpersonal Acceptance–Rejection Loneliness Scale (IPARLS; Senese et al., 2021), assessing the emotional component of feeling socially rejected or accepted on a scale from 1 (not at all) to 5 (completely); and three manipulation check questions assessing (a) how they felt during the Cyberball (accepted/rejected), (b) the percentage of throws received, and (c) to what extent they felt included by the other players (from 1 not at all to 5 completely). In the present study, IPARLS showed good reliability (Cronbach's α = .83), consistent with the validation of the Italian version (Senese et al., 2021).

#### Stimuli

Four female faces (i.e., RAFD090_02, RAFD090_12, RAFD090_26, RAFD090_27) were selected from the Radboud Face Database (Langner et al., 2010). For each face, there was a version with a neutral expression and a straight gaze (used in the Cyberball task and as the pre‐cue in the gaze‐cueing task), and two versions with an averted gaze to the left and to the right, with neutral or happy expressions. Based on available validation data (Langner et al., 2010), faces were selected for having 100% agreement for expression categorisation and for being similar on genuineness (Happy: *M* = 3.97, SD = 0.31; Neutral: *M* = 4.06, SD = 0.26, *t*(3) = 1.35, *p* = .269) but different on valence (Happy: *M* = 4.3, SD = 0.10; Neutral: *M* = 3.2, SD = 0.18, *t*(3) = 15.37, *p* < .001).

The four faces with neutral expressions and straight gazes were divided into two sets. For each group, half of the participants completed the Cyberball task with one set and the other half with the other set.

#### Cyberball task

The Cyberball task consisted of 30 trials. For the social inclusion group, the virtual players passed the ball to the participant approximately one‐third of the time, ensuring equal participation (10 times out of a total of 30 throws). For the social exclusion group, after passing the ball to the participant twice, the virtual players ceased passing the ball to the participant. This manipulation has been shown to effectively induce feelings of social exclusion, impacting participants' emotions and fundamental needs (Williams et al., 2000).

#### Gaze‐cueing task

The gaze‐cueing task consisted of 16 practice trials followed by 192 trials divided into three blocks of 64 trials each, resulting from the factorial combination of four faces (two seen in the Cyberball and two new faces), with two facial expressions (happy or neutral), two eye‐gaze directions (left, right), and two target positions (left, right). Gaze cues were non‐predictive of target location as on 50% of trials the target appeared at the same location looked at by the cue (i.e., valid cue), and on the other 50% of trials, the target appeared at the location opposite to that looked at by the cue (i.e., invalid cue). Target stimuli consisted of two letters, “L” and “T”, presented either on the left or the right side of the screen.

#### Experimental design

The experimental design was a 2 (Group: Inclusion, Exclusion) by 2 (Expression: happy, neutral) by 2 (Cue: valid, invalid) by 2 (Face: Seen, New) mixed‐factorial, with the first factor between‐subjects.

#### Apparatus

The two tasks were presented using E‐Prime Version 3.0 software (Psychology Software Tools, Pittsburgh, PA, 2012). Stimuli were presented on a 19‐inch LCD monitor (resolution 1920 × 1080, refresh rate 60 Hz). Responses were collected using a standard USB keyboard. Participants sat comfortably in front of a computer screen at a viewing distance of approximately 60 cm, and to prevent head movements for the gaze‐cueing task, a chin rest was used.

#### Procedure

**FIGURE 1:**
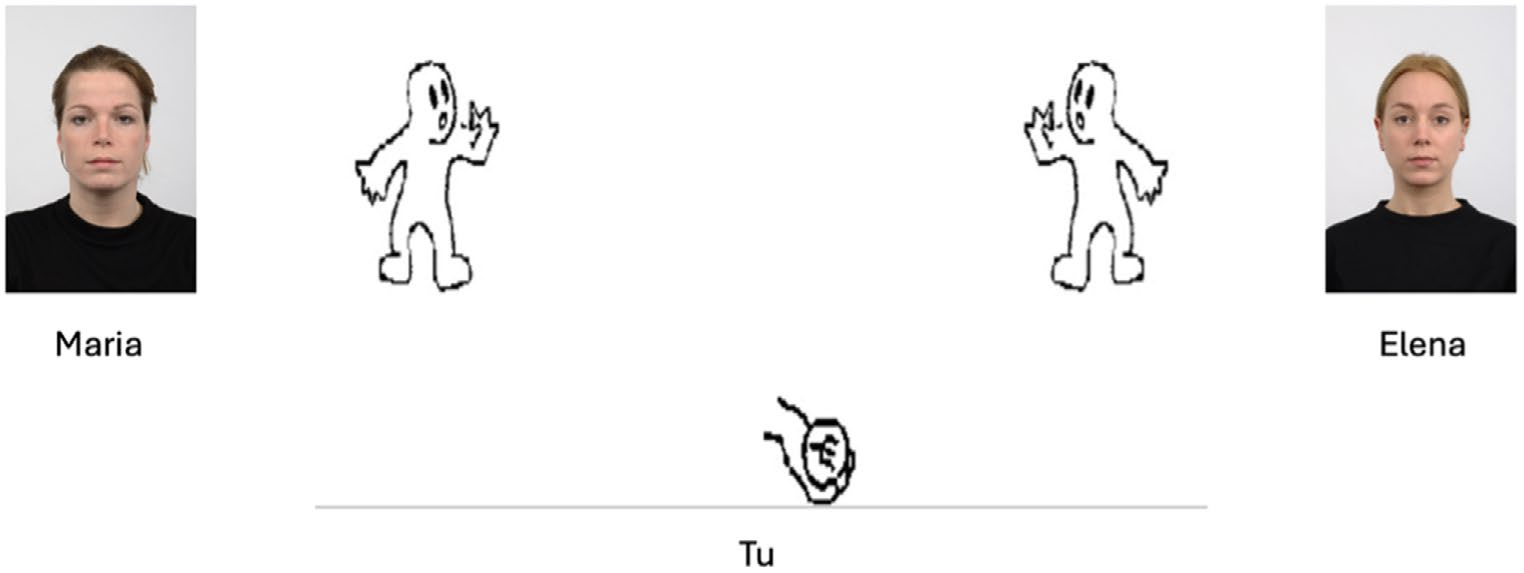
Example of a trial in the Cyberball task.

Upon arrival at the laboratory, participants provided their written informed consent, following which they completed the Cyberball task. Participants were informed that they would play a computerised ball‐tossing game with the two other virtual players. The “1” and “3” keys of the numerical keyboard were used to throw the ball to the other two players (1 for the player on the left and 3 for the player on the right of the screen). The experimenter launched the task and typed the participant's ID, which appeared onscreen as Tu (Italian for You) and the names of the two other players (Maria and Elena), which appeared below the two face pictures, measuring 6 × 7 cm, presented next to the silhouettes of the players, which measured 5 × 4 cm (see Figure 1).

Participants decided which player to pass the ball to without a time limit, and once passed, it took the ball 1 s to reach the other player. When the other two players passed the ball, the exchange lasted 2 s. In the exclusion group, participants received only 2 out of 30 ball passes, whereas in the inclusion group, all players received an equal proportion of ball passes (i.e., 10 out of 30 for each player).

At the end of the Cyberball task, participants completed the NTQ and the IPARLS questionnaires, as well as the three manipulation check questions, which were presented using Testable (Rezlescu et al., 2020). Once completed, the questionnaires, the experimenter launched the gaze‐cueing task. The task instructions appeared on screen, and participants were informed that they would see a face at the centre of the screen. The face would first look straight at them and then to the left or right, followed by a target letter presented to the left or right of the central fixation. Their task was to respond to the target letter as quickly and accurately as possible based on the letter identity. Participants were informed that gaze direction did not predict where the target appeared. Responses were given by pressing one of two keys (“1” and “5”) appropriately labelled and chosen to be perpendicular to the left and right locations. The key assignment to the target letter was counterbalanced across participants.

**FIGURE 2:**
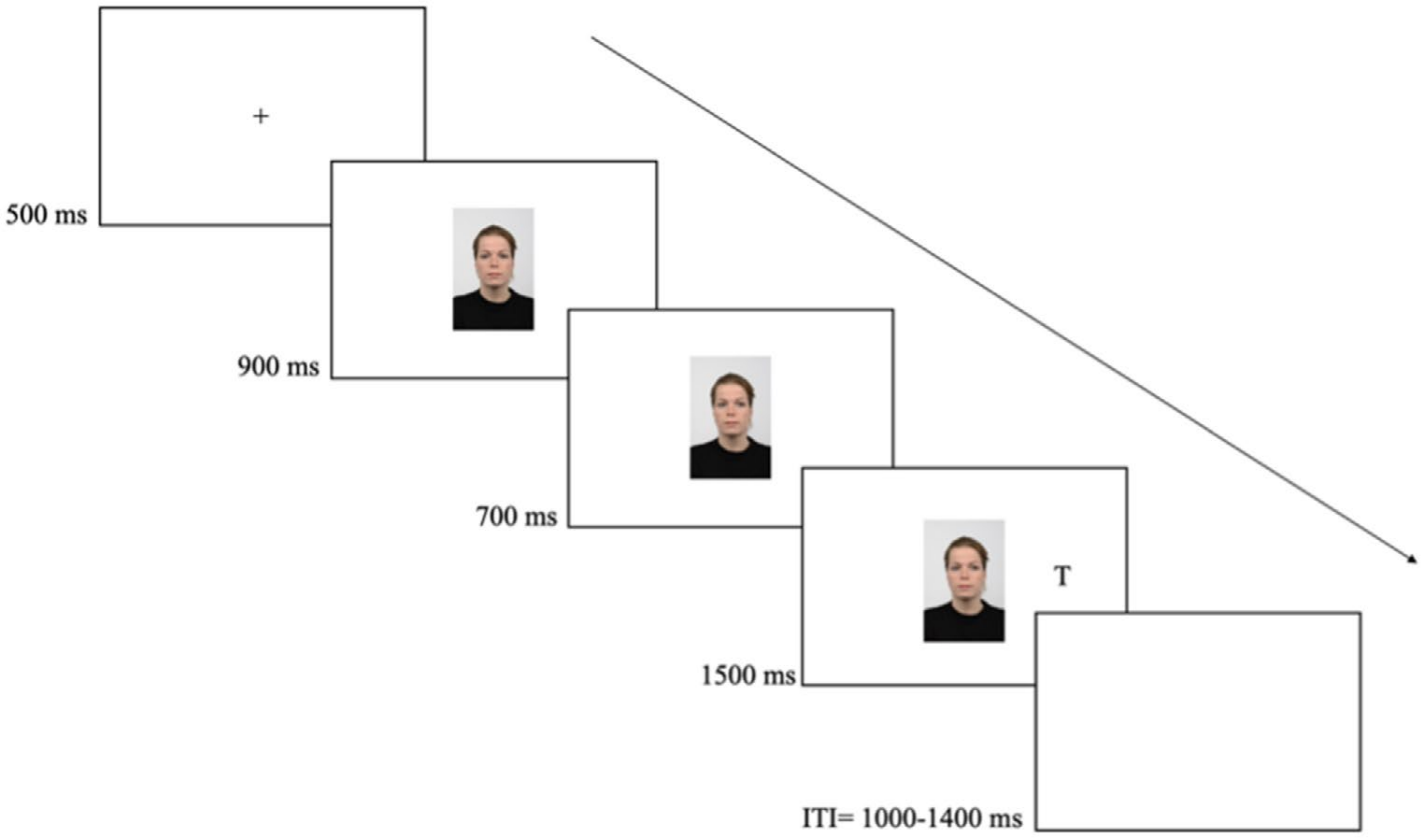
Trial sequence in the gaze‐cueing task. The example shows a typical trial with a neutral expression and a valid cue.

Each trial started with a centrally presented fixation cross (0.3 × 0.3 cm) for 500 ms, then a face looking straight ahead (pre‐cue) appeared for 900 ms, followed by the face looking left or right (face cue) for 700 ms. Both pre‐cue and cue measured 16 × 9 cm. Next, the target appeared either on the left or on the right of the screen (10 cm from central fixation) until a response was made or 1500 ms elapsed. The intertrial interval randomly varied between 1000 and 1400 ms in steps of 100 (see Figure 2).

Once the gaze‐cueing task was completed, and before being dismissed, participants (regardless of group) completed again the Cyberball task in the inclusion version again.

#### Data analysis

##### Preprocessing

Before conducting the analysis, the data were inspected for quality. For Study 1, two participants (one from each group) were excluded due to incomplete data. Trials with response times shorter than 200 ms were removed, as they were considered fast guesses (De Boeck & Jeon, 2019), accounting for 1.12% of the total number of trials. For Study 2 (see later), 1.7% of trials were removed as fast guesses.

##### Descriptive statistics

The proportion of correct responses was calculated to assess the accuracy performance. The mean and standard deviation (SD) of correct response times were computed to provide summary statistics for each experiment.

##### Cueing index

The cueing index was computed for each face (including those previously used in the Cyberball task and two novel faces) using the following formula from previous studies (e.g., Ramon et al., 2010; Salera, Boccia, & Pecchinenda, 2024; Salera, Yankouskaya, et al., 2024):

$$\left[\left(\mathrm{RT}\_\text{Invalid}-\mathrm{RT}\_\text{Valid}\right)/\left(\mathrm{RT}\_\text{Invalid}+\mathrm{RT}\_\text{Valid}\right)/2\right]\times 100.$$

This approach offers several advantages over raw difference scores. First, it normalises for individual differences in overall response speed (Adam et al., 1999; Baayen & Milin, 2010; Barthélémy & Boulinguez, 2002; Behrens et al., 2023; Wong et al., 2017). By expressing the cueing effect relative to each participant's mean RT, the index reduces the influence of global shifts in response speed that are unrelated to attentional processes. Second, the index is based on the symmetric percentage change (SPC), a scale‐invariant metric that facilitates more meaningful comparisons across participants and groups (Nuzzo, 2018). Finally, given that reaction time data are often positively skewed, the use of a proportional index helps to mitigate this issue and reduces the need for additional data transformations (Berry & Ayers, 2006).

To assess whether the magnitude of the cueing index in each condition significantly differed from zero, we conducted one‐sample *t*‐tests against a mean of zero. Additionally, we performed a Bayesian analysis to quantify the evidence for the cueing effects by calculating Bayes factors (BFs). BFs provide a ratio of the likelihood of the data under two competing hypotheses, offering a measure of evidence for one hypothesis over the other. For instance, a BF_10_ (indicating evidence for the alternative hypothesis over the null) greater than 3 suggests moderate evidence for the presence of an effect, while a BF_10_ less than 1/3 indicates moderate evidence for the absence of an effect. Values between 1/3 and 3 are typically considered inconclusive. In our analysis, we computed BFs using the JASP software, which employs a default Cauchy prior width of 0.707 for effect size under the alternative hypothesis, as recommended by Wagenmakers et al. (2010). We also used ANOVAs to assess for potential differences in cueing index between conditions and groups.^3^

##### Prevalence

We estimated the cueing effects by providing a full distribution of possible values for the effect's prevalence using a Bayesian framework. Bayesian prevalence focuses on detecting effects (in this case, gaze‐cueing effects) at the individual participant level and then inferring their occurrence in the wider population (Ince et al., 2021). We computed the Maximum A Posteriori (MAP) estimate representing the most probable value of the population prevalence given the observed data. It is derived from the posterior distribution, which combines prior knowledge and observed evidence. The MAP (i.e., the estimated most probable cueing effect value) serves as a point estimate of the prevalence of an effect in the population. To quantify uncertainty, Bayesian analysis also provides Highest Posterior Density Intervals (HPDIs), which indicate the range within which the true population prevalence is most likely to fall (i.e., the uncertainty of the most probable estimated cueing effect). The HPDI is a credibility interval that ensures a specified proportion of the posterior distribution (e.g., 96%) is contained within the interval, offering a nuanced view of data uncertainty compared to confidence intervals used in frequentist statistics. The Bayesian Prevalence Approach also enables us to compute between‐ and within‐group comparisons of the cueing effects (Ince et al., 2021).

## RESULTS

### Study 1

#### Ostracism manipulation

The summary statistics (Table 1) indicate that the experimental ostracism manipulation in this study aligns with the expected needs‐threat model. The results show increased distress in the excluded group across the four fundamental needs: belonging, self‐esteem, control, and meaningful existence, compared to the included group. Additionally, individual ratings of inclusion and acceptance further confirm this pattern.

**TABLE 1:** Mean (*M*) and standard deviation (SD) for Need‐Threat Questionnaire (subscales and total score) and for the manipulation checks.

Group	Belong	Control	Self‐esteem	Meaning	Tosses received	Rating of feeling included, 1 = not at all, 5 = completely	Rating of feeling accepted, 1 = accepted, 2 = rejected
Included	5.33 (2.21)	5.37 (1.68)	9.63 (3.04)	7.61 (2.60)	31.1 (12.6)	3.00 (0.82)	1.76 (0.43)
	Total need‐threat score = 27.9 (6.74)						
Excluded	8.89 (2.03)	9.76 (2.75)	11.9 (2.61)	10.7 (2.23)	7.24 (7.05)	1.67 (0.51)	1.02 (0.14)
	Total need‐threat score = 41.2 (7.34)						

Binary logistic regression was used to assess whether scores for belonging, self‐esteem, control, and meaningful existence validated the ostracism manipulation. The model significantly improved the prediction of group membership compared to the baseline (𝜒^2^(4) = 70.0, *p* < .001, *R* ^2^ = 0.55). Among the four fundamental needs, only control (*B* = 0.59, SE = 0.18, *Z* = 3.24, *p* < .001, Odds Ratio = 1.81) and belonging (*B* = 0.45, SE = 0.20, *Z* = 2.22, *p* = .02, Odds Ratio = 1.57) significantly predicted group membership. Self‐esteem (*B* = −0.14, *Z* = −0.97, *p* = .33) and meaningful existence (*B* = 0.28, *Z* = 1.67, *p* = .09) did not contribute to the model.

Additionally, an independent samples *t*‐test confirmed that participants in the included group reported feeling significantly more included (MD = 1.32, SE = 0.14, *t*(89) = 9.19, *p* < .001) and accepted (MD = 0.73, SE = 0.07, *t*(89) = 10.83, *p* < .001) than those in the excluded group. The effect size of these differences was large (Cohen's *d* = 1.92 and 2.27, respectively). These findings support the effectiveness of the ostracism manipulation in differentiating between the two groups, particularly through the impact on perceived control and belonging.

#### Descriptive statistics in cueing task

##### Accuracy

Participants in both groups were accurate in their responses, with accuracy ranging between 95% and 100% (see Material, Figure S1 and Table S1 for details). A Mann–Whitney *U* test indicated that participants in the excluded group were slightly more accurate compared to the included group (MD = 1.04, *U* = 589, *p* < .001, Rank biserial correlation = −.44; see details in Figure S2).

##### Response time

Mean response time and SD for each condition per group is reported in Table 2.

**TABLE 2:** Mean RT and (SD) for seen/new faces with different expressions (neutral, happy) in valid and invalid cue trials for excluded and included group.

	Seen				New			
	Neutral		Happy		Neutral		Happy	
	Valid	Invalid	Valid	Invalid	Valid	Invalid	Valid	Invalid
Included	524.55 (74.8)	521.99 (72.0)	516.95 (74.2)	533.28 (83.89)	518.89 (67.6)	532.63 (80.28)	522.25 (71.2)	531.68 (71.09)
Excluded	517.24 (96.9)	527.65 (86.2)	524.09 (89.7)	533.09 (99.51)	529.95 (91.2)	531.37 (97.93)	523.77 (85.3)	527.38 (84.92)

#### Cueing effects

**FIGURE 3:**
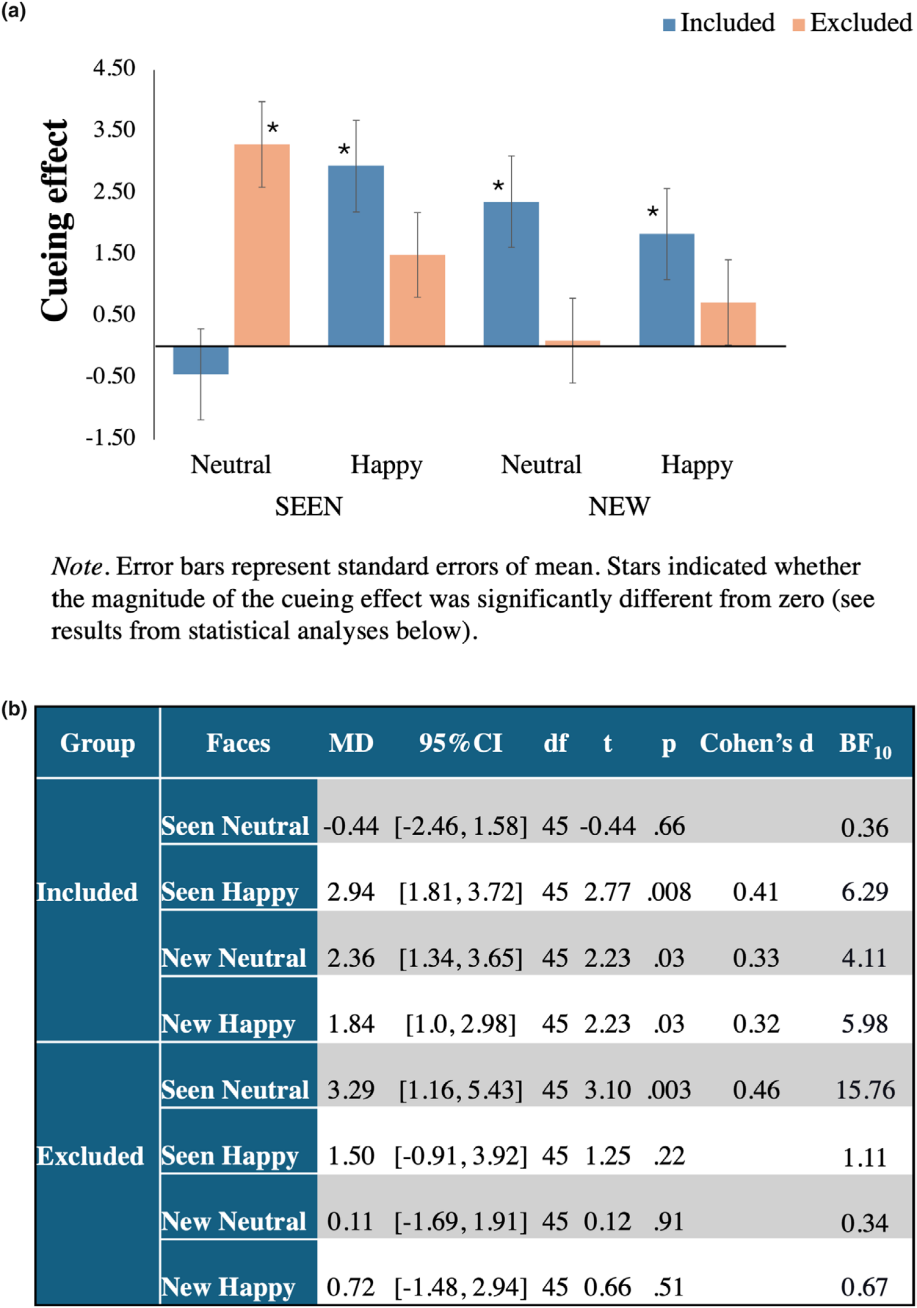
Cueing effects for seen/new emotional (happy and neutral) faces in included and excluded groups. (a) Visual representation of cueing effects; (b) Statistical results supporting the observed effects.

Results of a one‐sample *t*‐test against zero showed a stronger (i.e., positive value) cueing effect for seen happy faces for included participants, while excluded participants exhibited a stronger (i.e., positive value) cueing effect for seen neutral faces (Figure 3).

Results of the mixed‐factorial ANOVA with Group (2: included, excluded), Face (2: seen, new), and Expression (2: neutral, happy) showed that the main effects of Group, *F*(1,90) = 0.096, *p* = .76; Face, *F*(1,90) = 0.886, *p* = .35; and Expression, *F*(1,90) = 0.391, *p* = .53 were not statistically significant. Similarly, the two‐way interactions Expression by Group, *F*(1,90) = 2.21, *p* = .14; and Face by Expression, *F*(1,90) = 0.25, *p* = .62 were not statistically significant. In contrast, the Face by Group, *F*(1,90) = 5.58, *p* = .02, $\eta_{p}^{2}$ = .058 and the three‐way interaction, *F*(1,90) = 4.46, *p* = .037, $\eta_{p}^{2}$ = .047 were significant. Bonferroni‐corrected pairwise comparisons showed that the two‐way interaction was due to a significant difference in cueing effects for the two faces for the excluded group, (seen: *M* = 2.40, SE = 0.76 and new: *M* = 0.416, SE = 0.74), *p* = .022 but not for the included group, (seen: *M* = 1.25, SE = 0.76 and new: *M* = 2.10, SE = 0.74), *p* = .317. Bonferroni‐corrected pairwise comparisons for the three‐way interaction showed a significant difference in the gaze‐cueing effects between the two groups only for seen neutral faces, *p* = .012 (note that seen faces are of the excluder for the exclusion group and of the includer for the inclusion group). In contrast to Yang et al. (2024), socially excluded participants showed stronger gaze‐cueing effects for the excluder neutral face. None of the other comparisons were statistically significant, all *p*s > .25.

#### The prevalence of the cueing effects

Our next step was to quantify how typical or uncommon the cueing effects are in the population and the uncertainty around these estimates using Bayesian Inference of Population Prevalence (Ince et al., 2021). First, we estimated the prevalence of the true positive cueing effect (i.e., the proportion of the population we would expect to show a true positive cueing effect).

**FIGURE 4:**
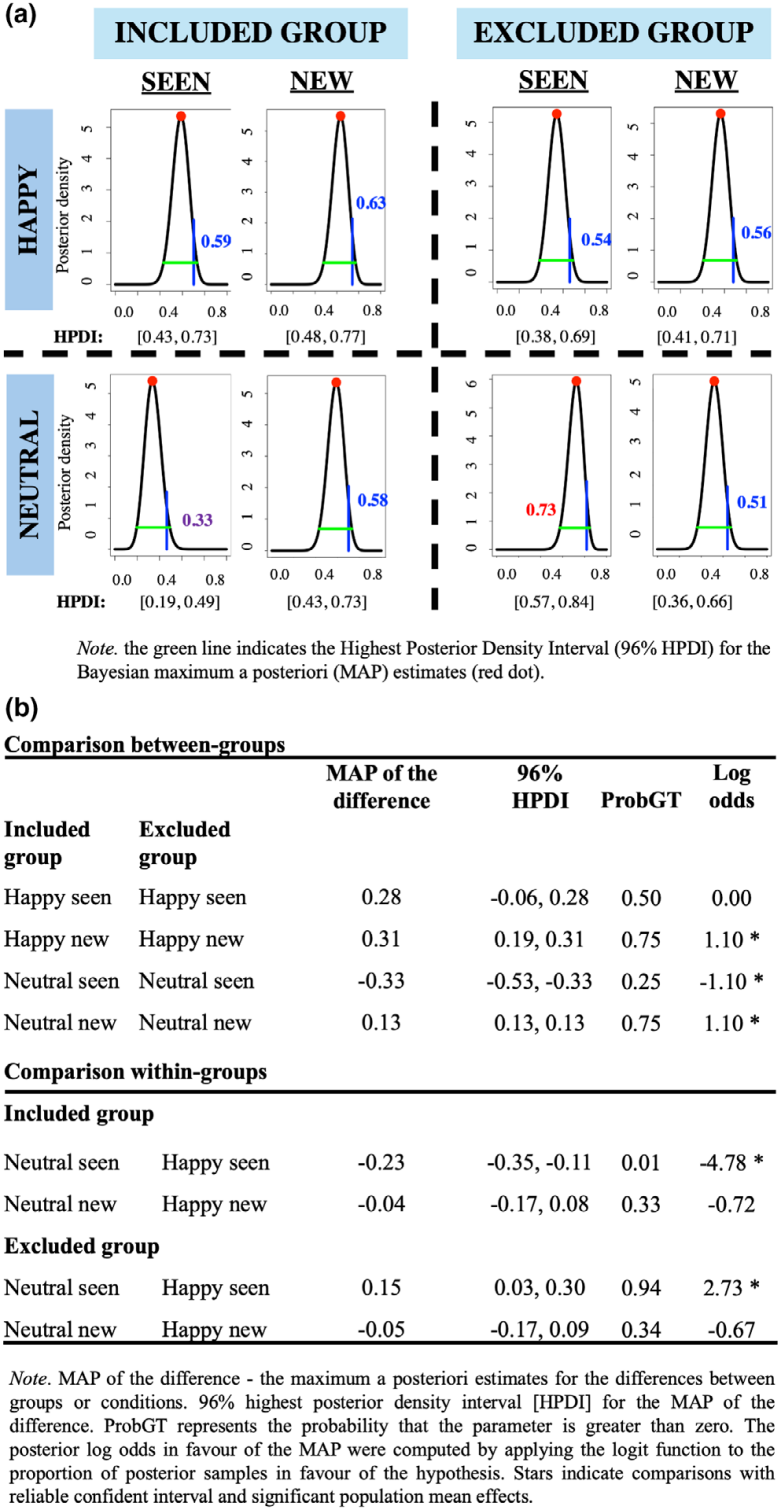
(a) Estimates of population prevalence of cueing effects in the included and excluded groups; (b) Estimated differences in prevalence between and within groups.

The results of this analysis showed that it is likely that only 33% of the population would show a true cueing effect in the included group for seen neutral faces, while in the excluded group, this effect is likely to be observed in 73% of the population (Figure 4a). Moreover, whereas in the included group, the prevalence of cueing effects for neutral seen faces was lower than for happy seen faces (59%), the opposite was true for the excluded group, for which the prevalence of cueing effects was higher for neutral seen faces compared to happy seen faces (54%) (Figure 4a). The prevalence of cueing effects for neutral and happy new faces was similar for the excluded (51% and 56% respectively) and for the included group (58% and 63%). Secondly, we estimated the differences in the prevalence of cueing effects between and within groups (Figure 4b).

Overall, although prevalence analysis showed some evidence of cueing effects in both groups, the percentage of individuals showing cueing with neutral previously seen (i.e., excluder for the social exclusion group and the includer for the social inclusion group) faces compared to happy previously seen faces (within‐group comparisons) was greater for the excluded group as opposed to the included group. This effect was not found for previously seen happy faces, where the percentage of individuals was similar for both groups. Therefore, social exclusion did not increase the number of individuals following the gaze of seen faces (i.e., the excluders) or new faces signalling affiliation (happy). Rather, social exclusion increased the number of individuals who followed the gaze of the excluders, showing a neutral expression.

#### The relationship between cueing effects

Our finding that exclusion selectively promotes social attention to excluders' neutral faces (i.e., stronger cueing effect for excluders' neutral faces) raises the question of whether the same participants who show this effect also exhibit differences in how they process happy faces. Since control and belonging were significant predictors of ostracism manipulation in our study (Results), we examined whether the relationship between cueing effects was influenced by these two fundamental needs. Particularly, those who feel included may not need to engage in social monitoring to the same extent, meaning that their attentional allocation to neutral and happy faces is less influenced by variations in control and belonging. This would explain why a relationship between neutral and happy cueing effects might emerge only in the excluded group. To test it, a partial correlation analysis was carried out to examine the relationship between cueing effects for neutral and happy faces while controlling for the effects of control and belonging.

For the Exclusion group, the results showed a negative correlation between cueing effects for neutral previously seen faces and happy previously seen faces, *r*(44) = −.38, *p* = .01, indicating that individuals who showed a larger cueing effect for neutral excluder faces showed a smaller cueing effect for happy excluder faces. There was also a positive partial correlation between cueing effects for neutral excluder faces and happy new faces, *r*(44) = .37, *p* = .01, suggesting that individuals who showed larger cueing effects for excluder‐neutral faces also showed larger cueing effects for new happy faces. In the Inclusion group, the results revealed no partial correlations between cueing effects (all *p*‐values > .05) (see Supporting Information S3).

Therefore, for the social exclusion group, individuals with larger gaze‐cueing effects for excluders' faces with neutral expression also had smaller cueing effects for signals of affiliation (happy expression) from the excluders but larger cueing effects for signals of affiliation from new individuals. This points to a pattern of social monitoring for the excluders and of sensitivity to affiliation from new individuals.

#### The social interpersonal relationships (IPARLS) and cueing effects

We also tested whether the magnitude of the cueing effects for the neutral and happy previously seen faces correlates with the degree of feeling sad as a result of the absence of adequate social interpersonal relationships. Our focus on the cueing effect in previously seen faces is motivated by the differences in these effects in both groups.

In both the included and excluded groups, IPARLS scores did not significantly correlate with any of the cueing effects (all *p*‐values > .05) (Supporting Information S4). This suggests that, within the included group, the degree of inadequate social interpersonal relationships did not systematically predict how individuals allocated attention to previously seen or new faces. In the excluded group, while exclusion itself may influence attentional biases, it does so independently of individual differences in broader social relationship difficulties.

### Study 2

This study aimed to investigate whether learning information about an episode of social exclusion affects joint attention with that individual. Past research has shown that having learned social information (i.e., social status, social disposition) about an individual just before the gaze‐cueing task affects following the gaze of that individual. Hence, Study 2 used a learning phase before the gaze‐cueing task to allow participants to learn about a fictive episode of social exclusion with that individual face, or about a fictive non‐exclusion (control) episode. This would allow assessing whether the effects observed in Study 1 rely on directly experiencing social exclusion immediately before or whether they can also occur based on just learned social information.

#### Participants

Ninety‐three university students (70 F; 23 M; age in years *M* = 20.75; SD = 2.4) who had not taken part in Study 1 were recruited in exchange for course credit. For the present study, using G*Power (Faul et al., 2009), we calculated the required sample size for a medium effect size of *f* = 0.25, with alpha error probability set at .05, power of 0.95, and six measurements using the *F*‐test for a repeated measure, within‐factors test (Faul et al., 2009). This analysis suggests a minimum of 28 participants in total would be required to achieve the power. All participants gave their informed consent, which was obtained according to the Declaration of Helsinki (1991). The study had received approval from the institutional review board (approval n. 0000867). Participants had normal or corrected‐to‐normal vision and were naïve to the purpose of the study.

#### Questionnaires

Participants completed the Italian version of the Interpersonal Acceptance–Rejection Loneliness Scale (IPARLS; Senese et al., 2021), and the Italian version of the Philadelphia Mindfulness Scale (PHLMS; Simione et al., 2022), which was included for exploratory purposes as it has been suggested that mindfulness may be beneficial in alleviating the impact of ostracism (Chen et al., 2022).

#### Stimuli

The stimuli were as in Study 1.

#### Learning task

The four female faces used in Study 1 were presented with a short description: two descriptions were designed to depict a social exclusion episode, and two were designed as controls. More specifically, participants learned:

- *Maria is in the WhatsApp group “Friends”, which has become silent. You find out that she has created another group without you (exclusion)*.
- *Marta is your friend, and you often go out together for dinner with friends. You find out that she has organised a dinner out without you (exclusion)*.
- *Anna attended the same school as you. You find out that she is enrolled in the Faculty of Economics (control)*.
- *Laura moved some time ago from another city with her parents. You find out that she is your neighbour ('control')*.

Association of the biographic information to each face identity was counterbalanced with different versions of the task. In the testing phase, participants were presented with the following prompts for each face:

- *She created another WhatsApp group without you*
- *She did not invite you to dinner with friends*.
- *She attended the same school as you*.
- *She is your neighbour*.

#### Gaze‐cueing task

The gaze‐cueing task was as in Study 1.

#### Experimental design

For the gaze‐cueing task, the experimental design was a 2 (Expression: Neutral, Happy), by 2 (Cue: valid, invalid) by 2 (Face: Excluder, Control) within subjects.

#### Apparatus

The learning task and the gaze‐cueing task were presented using E‐Prime Version 2.0 software. Stimuli were presented on a 19‐inch LCD monitor (resolution 1920 × 1080, refresh rate 60 Hz). Responses were collected using a standard USB keyboard. Participants sat comfortably in front of a computer screen at a viewing distance of approximately 60 cm, and to prevent head movements for the gaze‐cueing task, a chin rest was used.

#### Procedure

Upon arrival at the laboratory, participants provided their written informed consent, following which they completed the IPARLS and PHLMS questionnaires, which were presented using Testable (Rezlescu et al., 2020), followed by the learning task and by the gaze‐cueing task.

For the learning task, participants were presented with four trials, one for each face identity. They saw a face (16 × 9 cm) with a short description and were asked to memorise the face and the description for later recognition. When ready, participants moved to the next face by pressing the spacebar. This was followed by a testing phase, consisting of a block of 16 trials in which each of the four faces was presented four times with one of the four prompts (see above). Their task was to decide whether the prompts correctly applied to the individual by pressing one of two keys (the “s” and “n” keys) appropriately labelled as “si” (Italian for yes) and “no”. In the testing phase, each trial started with a central fixation cross (measuring 0.3 × 0.3 cm) for 1000 ms, followed by the face (measuring 16 × 9 cm) presented for 700 ms. The prompt then appeared on screen, and participants had no time limit to respond. Feedback was provided for 2000 ms.

These two phases – learning and testing – were repeated twice for a total of 40 trials (8 trials for learning information and 32 trials of testing). Participants completed the gaze‐cueing task only if they were able to correctly recognise the description for each of the four face identities. If participants did not reach 100% recognition accuracy, the procedure was repeated. For the gaze‐cueing task, the procedure was as in Study 1. Finally, at the end of the gaze‐cueing task, participants were again presented with each face identity and asked for the individuals' description they had learned. Almost all participants (96%) correctly retrieved the learned information for all four faces, whereas the others correctly retrieved the learned information for at least three faces.

#### Descriptive statistics

##### Accuracy

Response accuracy was high, ranging between 79% and 100% with an average of 96.7% across all conditions.

##### Response time

Mean response time and SD for each condition are reported in Table 3.

**TABLE 3:** Mean RT and (SD) for excluder/control faces with different expressions (neutral, happy) in valid and invalid cue trials.

Faces	Neutral		Happy	
	Valid	Invalid	Valid	Invalid
Excluder	517.34 (93.65)	525.77 (91.80)	513.85 (91.82)	523.21 (91.02)
Control	513.77 (95.47)	524.28 (93.02)	512.20 (88.63)	525.49 (92.06)

#### Cueing effects

**FIGURE 5:**
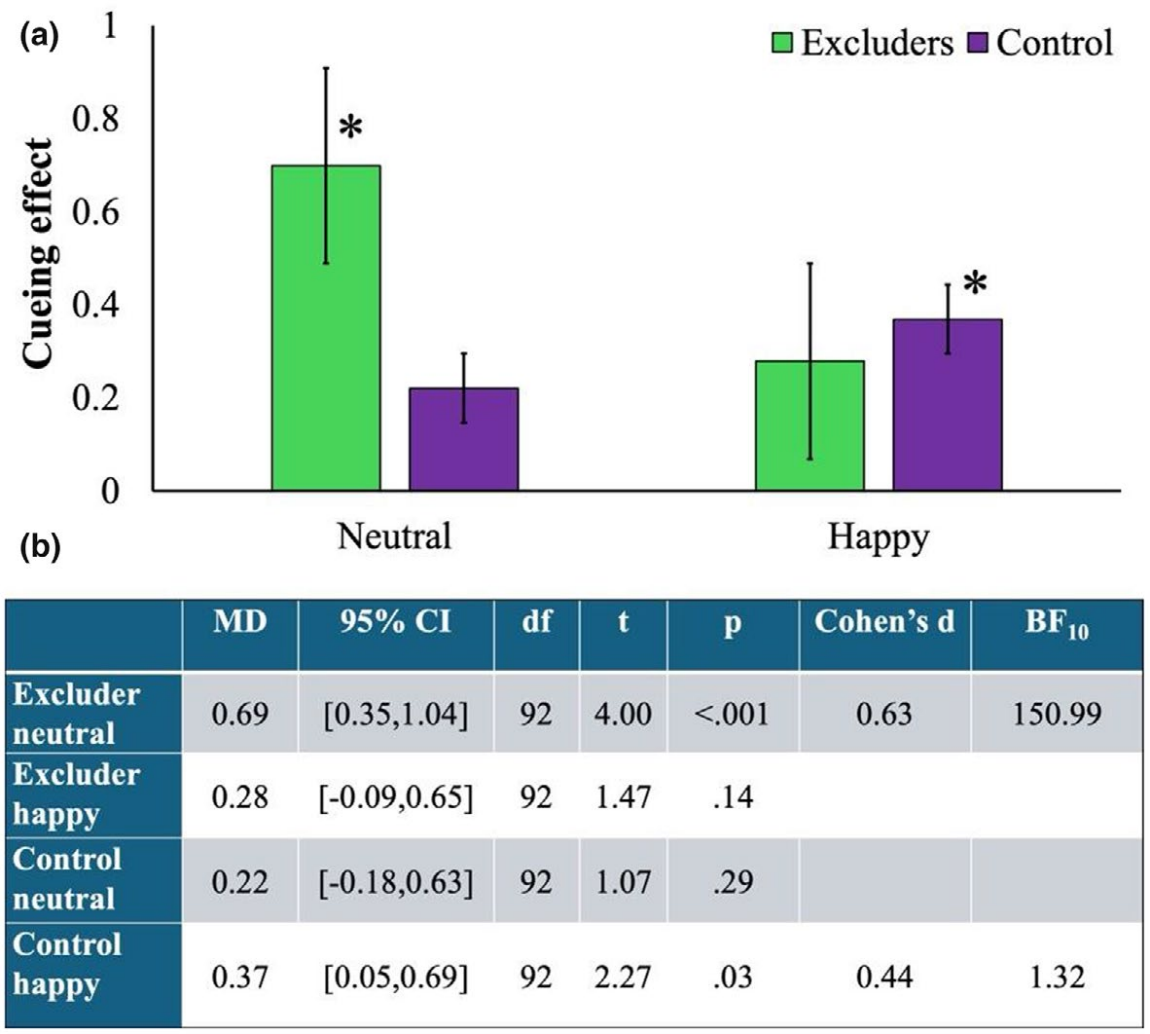
Cueing effects for excluders/control emotional (happy) and neutral faces for control and excluder faces. (a) Visual representation of cueing effects; (b) Statistical results supporting the observed effects.

A one‐sample *t*‐test was performed to test whether cueing effects were different from zero. The results showed that there were two cueing effects significantly greater than zero – for neutral excluder face and happy control face (Figure 5a). However, BF analysis provided strong evidence only for neutral excluder face, while there was no evidence for happy control face (Figure 5b).

Results of the repeated‐measure ANOVA with Face (2: excluder, control) and Expression (2: neutral, happy) showed that the main effects of Face *F*(1,92) = 1.30, *p* = .26 and Expression, *F*(1,92) = 0.57, *p* = .45 were not statistically significant. Similarly, the two‐way interaction Face by Expression, *F*(1,92) = 0.26, *p* = .11 was not statistically significant.

#### The prevalence of the cueing effects

**FIGURE 6:**
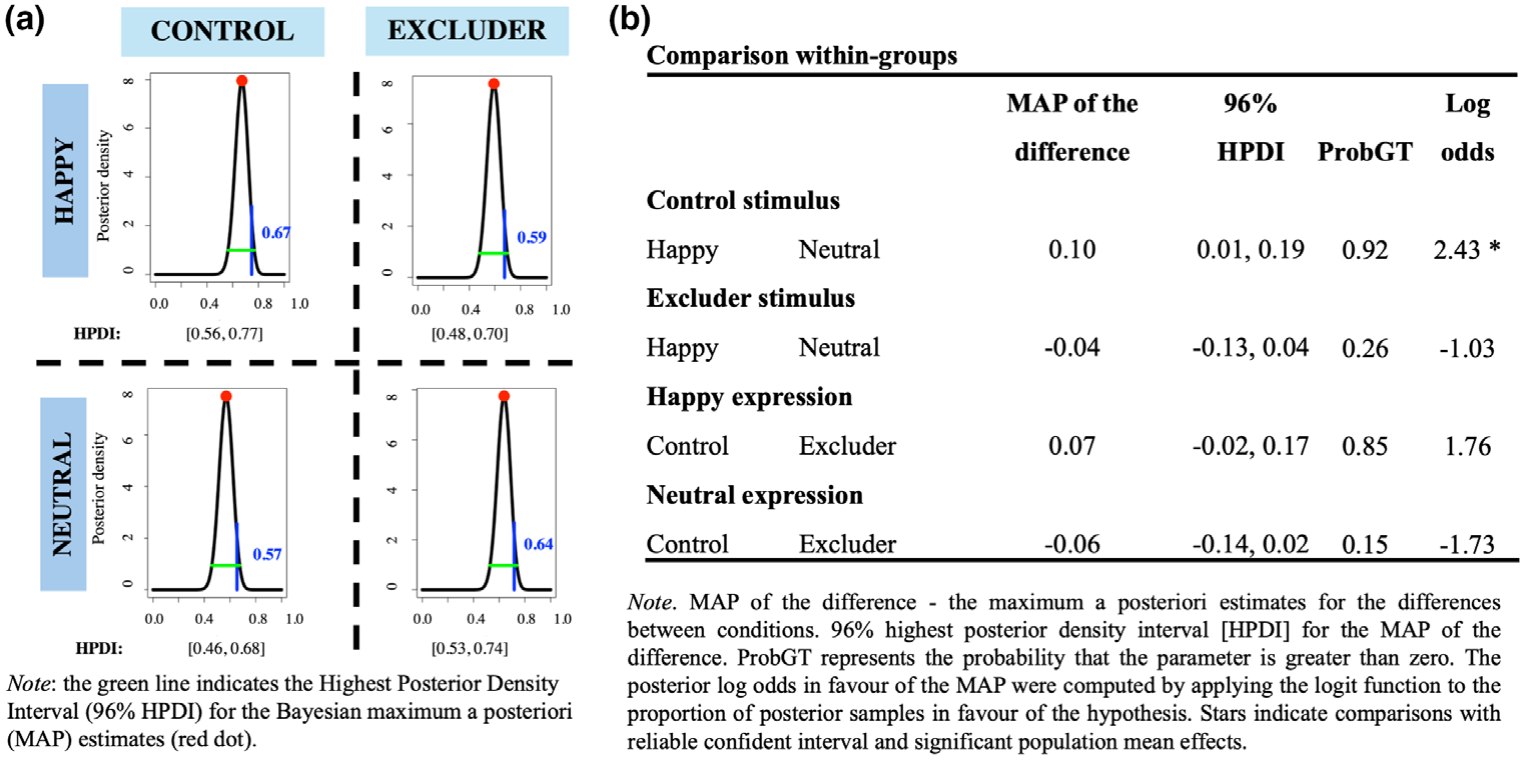
(a) Estimates of population prevalence of cueing effects in the Control and Excluder faces; (b) Estimated differences in prevalence of the cueing effects.

We next estimated the prevalence of true positive cueing effect and obtained the MAP together with measures of uncertainty for each condition. The results of this analysis showed that it is likely that 57% and 67% of the population would show a true cueing effect for neutral and happy control faces, respectively, while 64% and 59% of the population would show a true cueing effect for neutral and happy Excluder faces (Figure 6a). Importantly, although – similar to Study 1 – there was a higher prevalence of cueing effects for Excluder faces with neutral expression (64%) compared to happy expression (59%) (Figure 6b), the prevalence of cueing effects for Excluder faces did not differ across emotional expressions (Figure 6a). In contrast, for the control faces, the prevalence of happy faces (67%) was higher compared to neutral (57%) (Figure 6a).

#### The relationship between cueing effects

To examine the relationship between these effects, we performed a correlation analysis, which indicated a small, positive correlation between the cueing effects for the Excluder and Control happy faces (*r* = .29, *p* = .005). There was also a positive correlation between the cueing effects for Excluder neutral and happy expressions (*r* = .24, *p* = .02). All other pairwise correlations were non‐significant (*p* > .05) (see details in Supporting Information S5).

#### The social interpersonal relationships (IPARLS) and cueing effects

Similar to Experiment 1, we tested whether the magnitude of the cueing effects for the neutral and happy faces correlates with feeling sad due to a lack of adequate social interpersonal relationships. The analysis revealed two correlations between IPARLS and cueing effects for neutral control faces (*r* = .22, *p* = .03) and happy excluder faces (*r* = .23, *p* = .03) (Supporting Information S6).

In addition, we assessed the present‐moment awareness and acceptance, and we hypothesised that higher awareness would alleviate the impact of exclusion information on gaze‐cueing effects. Specifically, higher present‐moment awareness and acceptance may buffer against attentional biases toward socially threatening stimuli (Excluder neutral faces), leading to smaller cueing effects compared to individuals with lower awareness and acceptance. However, for control faces, cueing effects may remain stable regardless of mindfulness levels. Our results indicated that none of the cueing effects in this experiment were linked to awareness or acceptance (all *p* > .05) with BF analysis supporting the null hypothesis (see Supporting Information S7).

## DISCUSSION

We assessed the effects of being ostracised on following the gaze of the individual who socially excluded us or someone new when they are showing a happy or a neutral expression. This research question stems from the temporal need‐threat model (Williams, 2009), according to which the immediate reaction to being ostracised is to pay attention to the social situation and either attempt to be re‐included – therefore by paying attention to signals of affiliation – or to protect oneself from further exclusion. However, past studies did not assess gaze following when the excluder shows a signal of affiliation by smiling. Therefore, the present research fills this gap in the literature. In Study 1, we used the Cyberball task like Capellini et al. (2019), but we linked the avatars representing the two other players to specific face identities, which were then used in the gaze‐cueing task together with two new face identities, like Yang et al. (2024). Importantly, differently from past studies, here in the gaze‐cueing task, the face cues could show either a happy or a neutral expression, and differently from Yang et al. (2024) – each face first established eye contact with the participants – which communicates the intention to engage in an interaction – and then looked left or right with a happy or neutral expression.

Findings clearly show that our social exclusion manipulation with the Cyberball task was successful as participants in the excluded group reported fewer feelings of control and belongingness, two of the four basic needs affected by ostracism (Williams, 2009), and they also felt less included and accepted. Most importantly, excluded participants showed stronger gaze‐cueing effects when the face was that of the excluder with a neutral expression. Prevalence analyses, which inform us that the percentage of individuals following the gaze of faces already seen before (and yielding gaze‐cueing effects) showed that socially excluded participants had a specific pattern of gaze following that occurred less for the socially included participants. That is, most excluded participants (73%) showed stronger gaze‐cueing effects (i.e., greater positive values) only for seen neutral faces – that is, they followed the gaze of the excluders with a neutral expression. In contrast, included participants showed greater gaze‐cueing effects in all other conditions, except this one, for which gaze‐cueing effects were smaller (i.e., negative values) – only 33% of included participants showed cueing effects for seen neutral faces (that is, includer faces).

This finding may seem at odds with social exclusion reducing the gaze‐cueing effects for the excluder reported by Yang et al. (2024). However, in that study, the face was presented directly with averted gaze, and social exclusion affects the perception of the cone of gaze – that is, the degree of gaze deviation (e.g., Lyyra et al., 2017; Syrjämäki et al., 2020). This factor may have contributed to the observed effect. In contrast, having first presented the face with straight gaze (i.e., the pre‐cue) makes the gaze direction signal much clearer, as there is evidence that young children do not show gaze‐cueing effects when directly presenting a face cue with averted gaze, but they do when the face cue is preceded by a face with straight gaze (Pecchinenda & Petrucci, 2021). Therefore, having presented first a face that establishes eye contact by looking straight at the participant and signalling the intention to interact, followed by averted gaze, we may have reproduced a more ecological sequence, which minimises the difficulties of ostracised individuals in perceiving gaze direction. In fact, there is emphasis on the need to use more ecological settings to assess joint attention with the gaze‐cueing task (see McKay et al., 2023; Monachesi et al., 2025). Importantly, whereas our findings are at odds with Capellini et al. (2019), they align with Wilkowski et al. (2009, Study 2), who also presented a face with a straight gaze first. It is interesting that, like in Wilkowski et al. (2009, Study 2), our excluded participants reported lower control and belongingness – two of the four basic needs affected by ostracism. This suggests that the effects of social exclusion on following the gaze of others are shaped by changes in these two basic needs. The present finding adds to and extends the available evidence by showing that social exclusion, by affecting belongingness, enhances gaze cueing toward the specific individual who ostracised us. This occurs when that person first signals an intention to interact by looking at us (i.e., straight gaze) with a neutral facial expression and then looks somewhere else. Why would an excluder face with a neutral expression produce stronger gaze cueing than an including face, and why is this not just “more attention” to that face? One possibility is that neutral faces, unlike happy faces, which clearly signal social acceptance and affiliation, are more ambiguous. In fact, under normal circumstances, neutral faces can be interpreted as a potential threat (see Kauschke et al., 2019), and social exclusion may increase the chances of doing so, especially when neutral expressions occur among happy ones (Chen et al., 2017; Syrjämäki & Hietanen, 2019). For example, feeling left out can make someone more sensitive to signs of rejection, and as a result, they might be more likely to see neutral faces in a negative light. This might be a way of staying alert to possible further rejection, but it can also lead to misunderstanding social situations that are neutral. The neural overlap between social and physical pain (Eisenberger & Lieberman, 2004) may help explain why ambiguous social signals become more salient following social exclusion, suggesting that the brain engages an alarm system sensitive to potential further rejection.

Another important finding is that social exclusion does not affect how many individuals follow the gaze (and showing gaze‐cueing effects) of happy faces already seen before. Moreover, the prevalence of cueing effects for neutral and happy new faces was similar for the excluded (51% and 56% respectively) and for included (58% and 63%) individuals, and comparisons of both between and within participants suggest that these differences did not fall within the reliable confidence interval.

At the population level, more socially excluded individuals follow the gaze of the excluder with a neutral expression, but the number of individuals who follow the gaze of someone new, smiling is similar for excluded and included individuals, pointing to a social monitoring mechanism with greater attention to socially relevant information (Pickett et al., 2004). This is a very functional pattern of social monitoring for non‐affiliation signals from the excluder (neutral – perhaps interpreted as threat) and for affiliation signals (happy) from new individuals as well as from the excluder. Interestingly, this is a bit different from what is present at the individual level, as our correlation analysis suggests that when social exclusion affects the individual's needs for control and belonging, the gaze cueing for the excluder neutral face is negatively related to the gaze cueing for the excluder happy face, but it is positively related to that for a new, happy face. This pattern aligns with the social monitoring system theory (Pickett & Gardner, 2005), as individuals who experience ostracism and need to re‐establish belonging become more receptive to novel opportunities for affiliation (i.e., from happy faces of new individuals) present in the environment but less to cues of affiliation from the excluder, while still being attuned to cues associated with rejection or ambiguity (i.e., a neutral expression) from the excluder. From a behavioural perspective, it may reflect an adaptive mechanism such as monitoring for a known social threat and being careful about an affiliative signal from a known social threat (i.e., the excluder) while being open to more positive opportunities for social connection (i.e., from new individuals). Finally, the lack of relation between the IPARLS scores and social exclusion suggests that the effect we observe is the “immediate” effect of social exclusion, whereas self‐reports of social rejection—as assessed by the IPARLS – may take more time to become evident.

To sum up, the immediate effects of social exclusion on joint attention are twofold: (a) it tunes us to possible signs of threat from the individual who ostracised us, (b) while still motivating us toward possible signs of re‐affiliation from new individuals.

Having established what the immediate effects of being socially excluded are when seeing again the individuals who perpetrated the ostracism and how it affects our propensity to follow their gaze, in Study 2, we assessed whether these effects also occur when episodic information about the faces has just been learned. Therefore, we varied the social information about different individuals, and we then used their faces in the gaze‐cueing task. In line with evidence showing that dispositional information learned about faces affects the gaze‐cueing effect (i.e., Carraro et al., 2017; Dalmaso et al., 2012, 2014; Hudson et al., 2012), in Study 2 the learned social information did affect the gaze‐cueing effects, but it did so in a way that is different from Study 1. More specifically, in keeping with the findings of Study 1, there was strong evidence of cueing effects for neutral excluder faces (i.e., values greater than zero), whereas the evidence of cueing effects for happy control (new) faces was not substantiated by Bayesian results. In our Study 2, there are stronger cueing effects for excluder faces is in line with findings of stronger gaze‐cueing effects elicited by persons performing anti‐social behaviours (Carraro et al., 2017; Hudson et al., 2012). It is, however, important to note that prevalence analysis clearly shows that the percentage of individuals following the gaze of neutral faces is similar for excluders (64%) and control faces (57%). Finally, our correlation analysis shows that at the individual level, there is a positive association between gaze‐cueing effects for excluder and control faces showing signals of affiliation (happy expression). In contrast, there is a positive association between gaze‐cueing effects for excluders' happy and neutral faces. This correlation is negative in Study 1, which seems to be specific to having experienced immediately before, social exclusion from an individual. Finally, and again differently from Study 1, cueing effects for neutral control faces and for excluder happy faces correlated with Interpersonal Acceptance–Rejection Loneliness scores but not with the mindfulness scores. These differences point to experiencing social exclusion as a necessary condition for the dual effect on social attention observed in Study 1 to occur. Altogether, our findings offer new insights into how most individuals respond to social exclusion in the first stages following the experience. Rather than simply withdrawing, socially excluded individuals appear to remain socially engaged by monitoring both for potential known social threats (i.e., the excluder) and for novel opportunities to reconnect. This dual pattern – attention to threat (from the known excluder) and to affiliation (from novel individuals) – is specific to experiencing social exclusion, and it may help explain how short‐term experiences can contribute to longer‐term difficulties. Vigilance toward ambiguous social signals may, over time, feed into cycles of misinterpreting, hyper‐sensitivity to social threat, and feelings of loneliness. At the same time, continued attention to affiliative cues suggests that the motivation to reconnect remains intact.

### Constraints on generality

The sample of the present study represents the typical population of our university of western, educated, industrialised, rich, and democratic (WEIRD) samples. Although, according to theory, the immediate, *reflexive* response to social exclusion is independent of individuals' characteristics, what may vary between is the response at later stages of the social exclusion. Accordingly, contextual factors such as being part of an individualistic or collectivistic society may affect the way we interpret and respond to social exclusion. This being considered, our findings indicate that the immediate effect of social exclusion consists in monitoring for both potential social threats from the known excluder as well as for novel opportunities to reconnect. This finding prompts a direction for future work: exploring how interventions accessing positive social cues might reduce the longer‐term emotional costs of exclusion, particularly in people vulnerable to chronic loneliness. In addition, our findings show that not everyone responds to social exclusion in the same way. While most excluded individuals followed the gaze of the excluder with a neutral expression, a small proportion did not. Exploring what personal characteristics (e.g., trait loneliness, emotion regulation, rejection sensitivity) might shape these immediate and potentially lasting responses to exclusion can help identify those most at risk of the effects of exclusion and to develop interventions to support social re‐engagement. One direction for future work is to examine how these attentional patterns evolve over time. For example, whether individuals who show greater attention to threat‐related cues immediately after exclusion go on to experience greater social disconnection or loneliness at later points. Importantly, our findings also show that even in the face of exclusion, people remain tuned in to social cues and open to connecting with new people—an important foundation on which to build more inclusive social environments.

## AUTHOR CONTRIBUTIONS

**Ala Yankouskaya:** Methodology; data curation; formal analysis; visualization; writing – review and editing; investigation. **Claudia Salera:** Software; writing – review and editing; data curation; investigation. **Marianna Constantinou:** Data curation; formal analysis; writing – review and editing. **Anna Pecchinenda:** Conceptualization; investigation; methodology; software; data curation; funding acquisition; writing – original draft; project administration; resources; supervision; formal analysis.

## Supporting information


Data S1:

## Data Availability

All primary data and analyses scripts are publicly available at the Open Science Framework – OSF – at the link: https://osf.io/tmf4p/?view_only=a90a5699037342d9a6b1075e515069ac.

## Footnotes

^1^
Although the expression “following the gaze of others” is often used in studies recording eye movements, it is not exclusive to these studies. Here, it is used as in McKay et al. (2023) to denote the dynamic of a social interaction from establishing eye contact with the pre‐cue with direct gaze to following the direction of eye gaze of the other person, and it refers to an attention shift via the gaze cue stimulus, which is considered to have occurred when cueing effects (faster responses to validly cued targets than to invalidly cued targets) are observed. Also, where some make a distinction between shared attention and joint attention, here the two terms are used interchangeably (see Stephenson et al., 2021).

^2^
This is because we are not interested in the difference, but in the magnitude of these differences.

^3^
Although we used a different analysis strategy, we thank an anonymous reviewer who also suggested reporting the results of the ANOVA because it allows assessing whether the magnitude of the cueing index varied across experimental conditions or groups, which cannot be addressed with one‐sample tests alone, and it may be useful to make direct comparisons to past studies.
