# Supplementary material for: Effects of social exclusion on following the gaze of others
Source: Br J Psychol. 2025 Oct 6;117(1):406–28. doi: 10.1111/bjop.70034 (PMC12783868; doi:10.1111/bjop.70034)
Supplement: Supplementary file 1 — Data S1: [file BJOP-117-406-s001.docx]

**Supplementary Material**

***S1. Accuracy performance***

Fig S1. Accuracy performance per group. The error bars represent SEM.


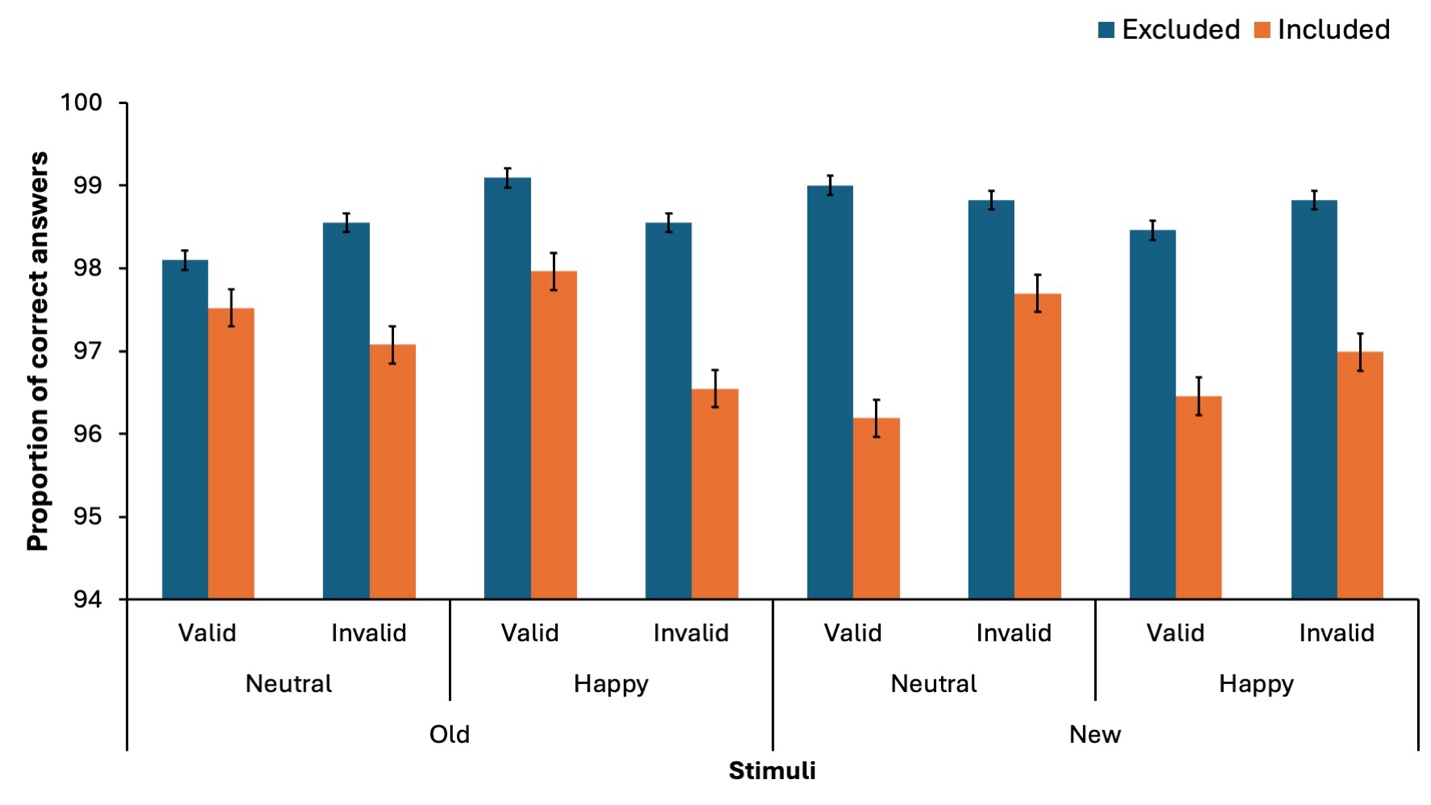


Table S1. Mean accuracy per condition in each group.

|  | **Old** | | | | **New** | | | |
| --- | --- | --- | --- | --- | --- | --- | --- | --- |
|  | **Neutral** | | **Happy** | | **Neutral** | | **Happy** | |
|  | Valid | Invalid | Valid | Invalid | Valid | Invalid | Valid | Invalid |
| Excluded | 98.10 | 98.55 | 99.09 | 98.55 | 99.00 | 98.82 | 98.46 | 98.82 |
| Included | 97.52 | 97.07 | 97.96 | 96.54 | 96.19 | 97.70 | 96.45 | 96.99 |

Fig S2. Overall mean accuracy (included and excluded groups)


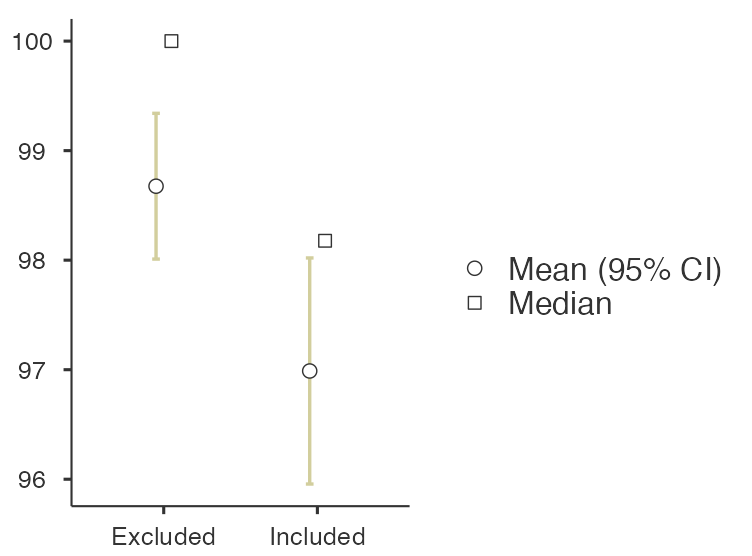


***S2. Response time performance***

Fig S3. Mean response time for each condition per group. Error bars represent SEM.


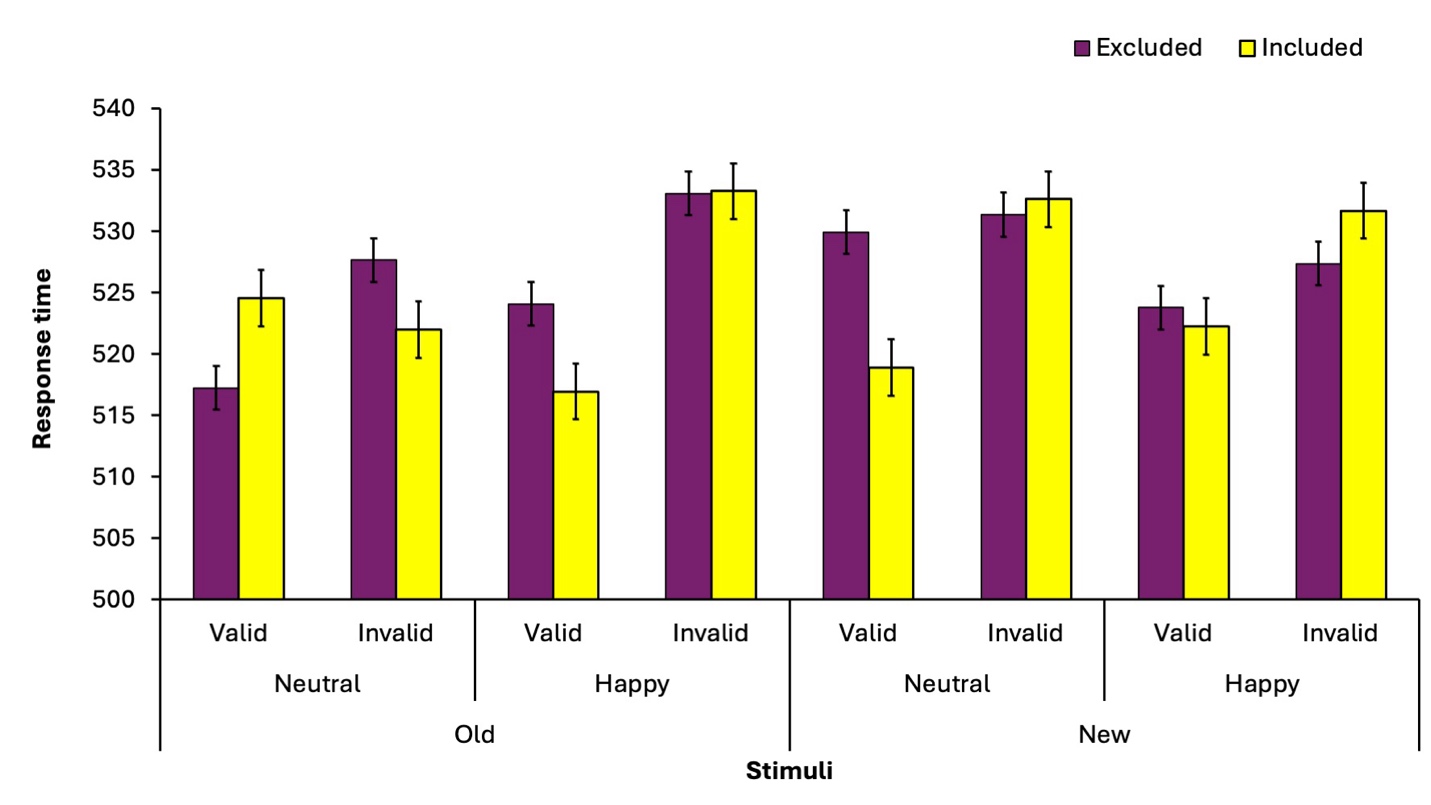


**S3. Partial correlation**

**
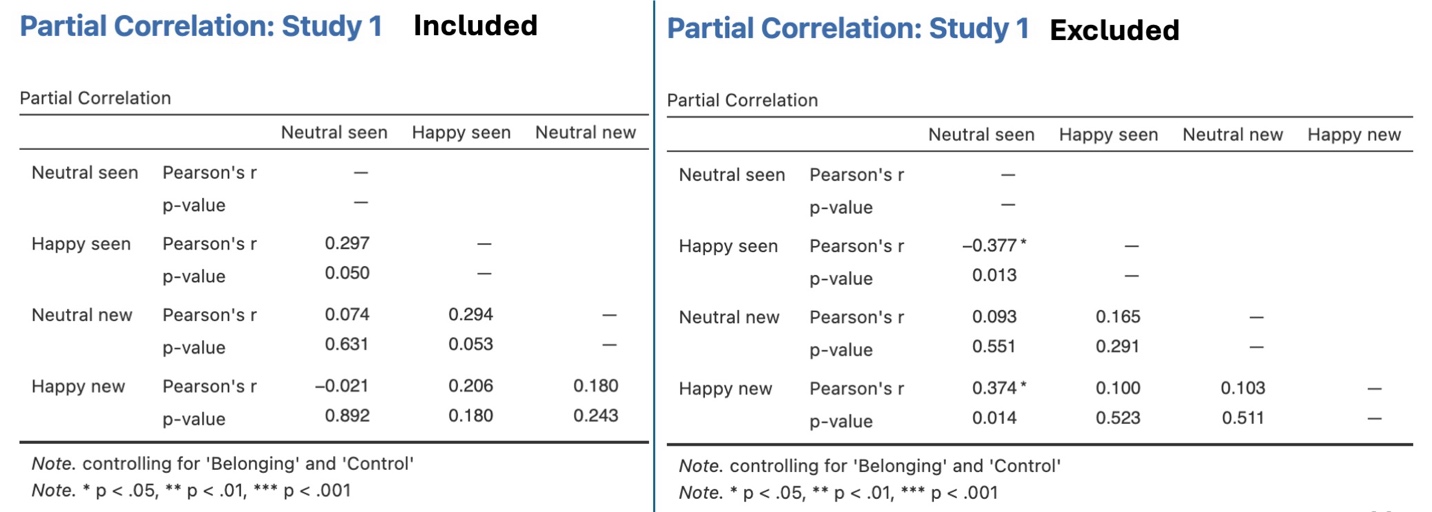
**

**S4. Correlations with IPARLS**

**
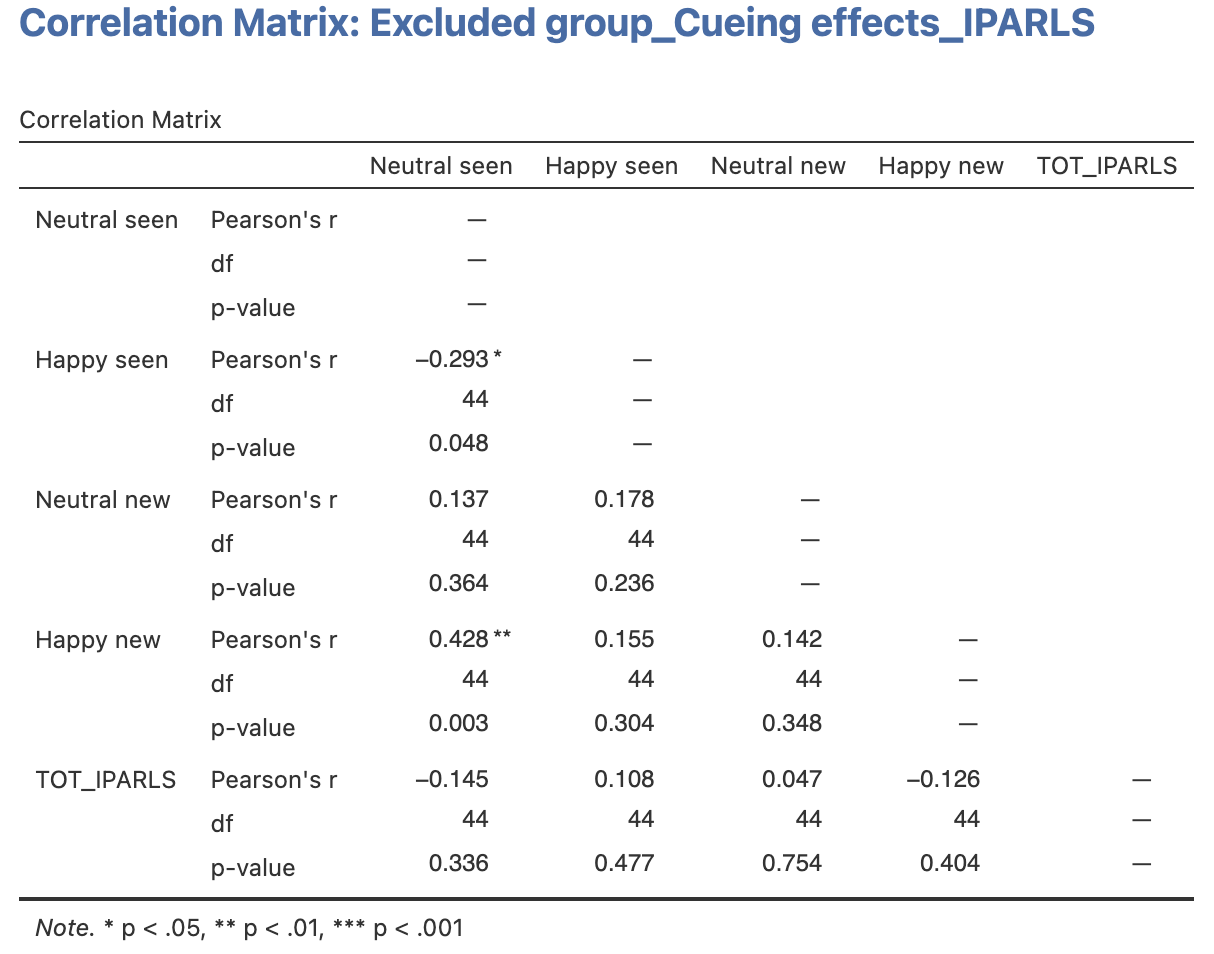
**

**
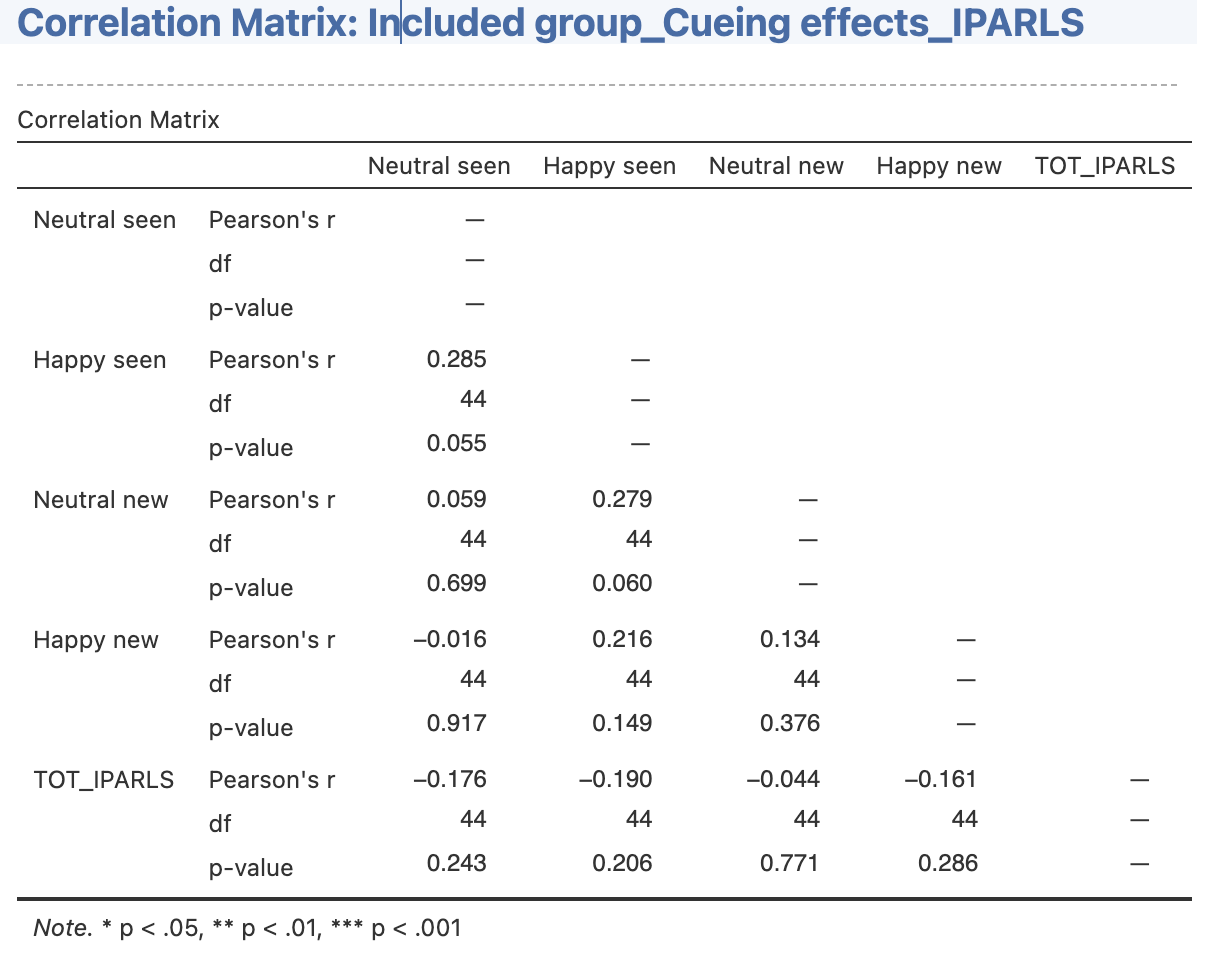
**

**S5. Correlation (cueing effects in Study 2)**

**
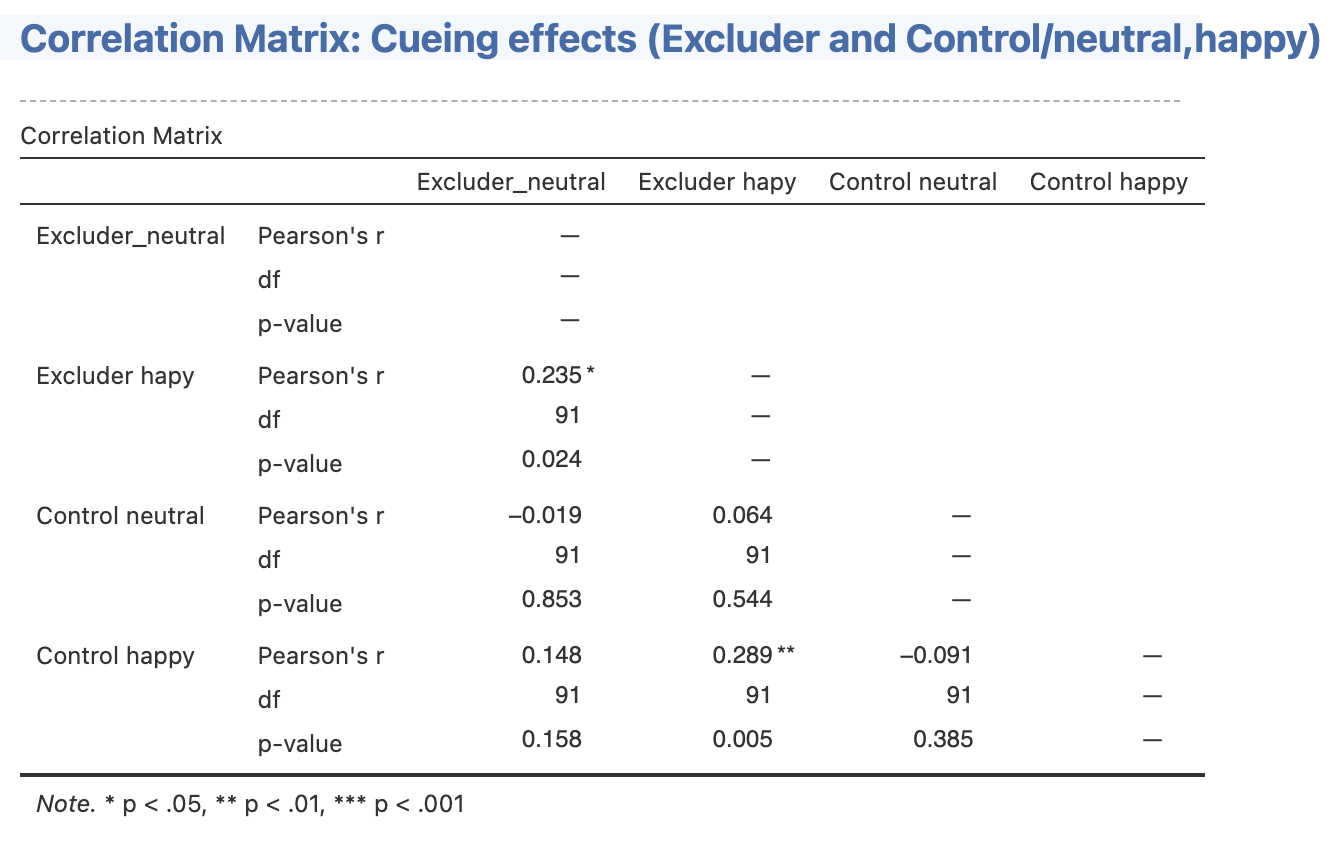
**

**S6. Correlation (cueing effects and IPARLS in Study 2)**

**
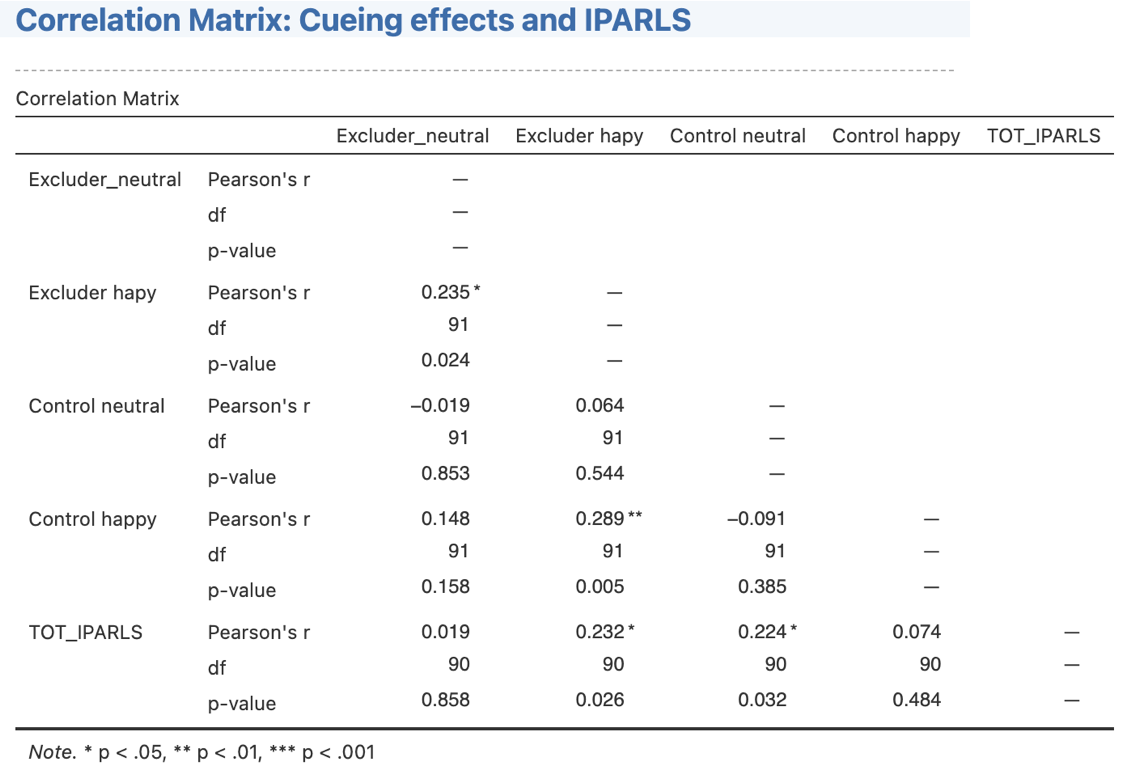
**

**S7. Correlation between cueing effects, awareness and acceptance
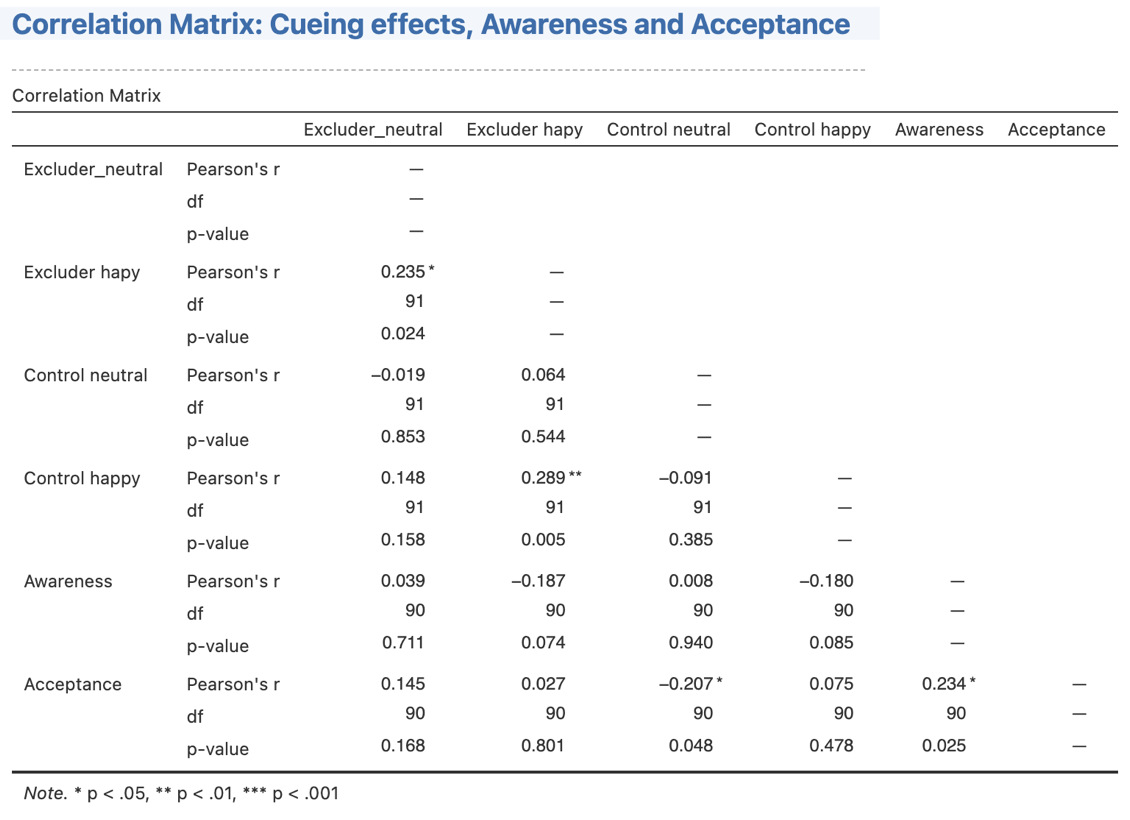
**

**
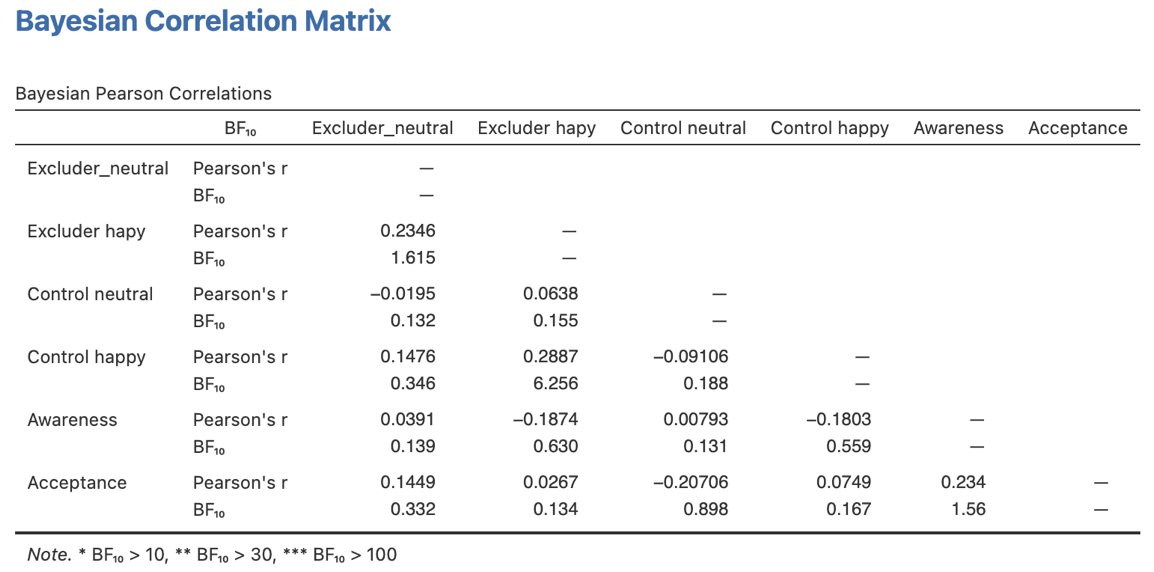
**
